# The Ideals of Facial Beauty Among Chinese Aesthetic Practitioners: Results from a Large National Survey

**DOI:** 10.1007/s00266-018-1241-8

**Published:** 2018-10-04

**Authors:** Souphiyeh Samizadeh

**Affiliations:** 1 Great British Academy of Aesthetic Medicine, London, UK; 2 King’s College London, London, UK; 3 Queen Mary University of London, London, UK; 4 Shanghai Jiao Tong University, Shanghai, China

**Keywords:** Ideals of beauty, Chinese facial aesthetics, Facial shape, Jaw angle, Chin, Facial profile, Chinese aesthetic doctors, Chinese dermatologists, Chinese plastic surgeons

## Abstract

**Abstract:**

As the demand for cosmetic procedures increases, the importance of patient-centred care in this field becomes more prominent. The aesthetic practitioners’ ideals of beauty, in addition to their knowledge and perception of patients’ ideals of beauty and expectations, are important during doctor–patient communication. These are important in strengthening practices of patient-centred communication and treatment. This study was conducted to investigate ideals of facial beauty among Chinese aesthetic practitioners. A questionnaire with simple sketches of facial features was given to aesthetic practitioners in Chinese cosmetology hospitals and clinics to assess aesthetic practitioners’ ideals of beauty and their preferences for facial shapes, facial profile, nose and lip shape, jaw angle, and chin shape. A total of 596 surveys were completed. This survey revealed that Chinese aesthetic practitioners preferred a heart/inverted triangular facial shape with a reduced lower face height, a straight and small nose, as well as lips that are full medially and taper off laterally with well-defined borders and Cupid’s bow. An obtuse jaw angle for women and a square well-defined jaw angle for men, and a round and pointy chin for both women and men were the most preferred. A majority (66.7%) of the respondents said they would have plastic surgery. However, if given the choice 82.9% indicated they would opt for non-surgical procedures. Finally, a clear majority (90.5%) believed that being beautiful would improve their daily life. The results were then compared to a similar previous study in which the same ideals of beauty were investigated among Chinese laypersons. This information will help the aesthetic professionals to understand their patient’s requests and expectations better and therefore aid in offering and providing treatments that are in line.

**Level of Evidence V:**

This journal requires that authors assign a level of evidence to each article. For a full description of these Evidence-Based Medicine ratings, please refer to the Table of Contents or the online Instructions to Authors www.springer.com/00266.

## Introduction

Multiple factors have contributed to the increased demand for surgical and non-surgical cosmetic procedures among both Caucasians and Asians. These factors include increased cultural acceptance of cosmetic procedures, growing ethnic populations, and media emphasis on personal appearance [1]. The American Academy of Cosmetic Surgeons’ 2006 Consumer Perception Survey reported that 46% of surveyed consumers would prefer cosmetic surgery to other goods and services including expensive vacations and high-end vehicles. Escalating economic power among ethnic minority populations in the USA and Europe has created an additional and potentially lucrative market for interested plastic surgeons. For example, the Plastic Surgery Statistics Report for 2016 found a 6% increase in cosmetic demographics of the Asian–American population with 1,154,084 procedures carried out [2]. In addition to the above, other factors such as scientific advances, improved technology and devices, and reduced cost of treatments have also contributed to the rising demand for cosmetic procedures [3].

The motivations and desires of patients seeking cosmetic procedures seem to be the same globally, including reducing the appearance of ageing, body contouring and definition, improving and enhancing symmetry, and changing tissue volume [1]. However, in general, it is believed that the majority of patients who seek cosmetic procedures want to maintain their ethnic identity and do not want to lose important facial features that exhibit racial characteristics [3, 4]. Therefore, it can be seen that the perception and ideals of beauty have multifactorial foundations including genetic and environmental factors [5].

There is a growing Chinese diaspora with reported number of over 40.3 million overseas Chinese in 2011 [6]. In addition, nowadays, more Chinese people are seeking medical and cosmetic treatments abroad [7]. Given this situation, aesthetic practitioners who treat Chinese patients in China or other countries would benefit from understanding the ideals of beauty among laypersons and also understanding how their own perceptions and ideals may vary from those of their patients.

The aim of this study was to understand the perception of Chinese aesthetic practitioners including ideals of beauty and the effect on treatments offered, requested, and carried out, and to compare this to the layperson’s point of view. The questions to be addressed are: Does the perception of beauty among aesthetic doctors and professionals match that of patients and does doctors’ perception of beauty affect the treatments they offer to their patients and carry out?

## Methods

A questionnaire was designed to determine the ideals of beauty for various facial features. Simple sketches were used and a total of 18 questions. The survey questions included demographic information (age group, gender, education, and where in China they came from), and evaluation of opinions about facial features including facial shape, facial profile, nose, lip and chin shape, and jaw angle. In addition, the survey participants were asked about the likelihood of them undergoing cosmetic surgery, and their preference for surgical and non-surgical aesthetic procedures if given both options. In addition, the participants were asked if they thought “being beautiful” would improve their quality of life. Each group of sketches were placed in a random order to avoid the potential for biasing participants’ decisions. Multiple choice questions and a random sample frame were used.

The author visited hospitals specializing in cosmetic treatments across China. The questionnaires were given to aesthetic practitioners in paper format. Cosmetic doctors, plastic surgeons, dermatologists, and consultants were included. (Note: in China, beauty consultants are mainly non-healthcare professionals who are the first line of patient interaction and communication. They carry out facial assessments on patients, offer treatment plans, and suggest treatment products or devices and filter patients prior to doctors seeing the patients and carrying out the treatment.) The survey collected the same information as in a previous study conducted among Chinese laypersons [8]. Each participant was given only one questionnaire. The questionnaires were distributed on different days in different hospitals, and 10–20 min were allowed for completion prior to being collected. The surveys were distributed and collected in the presence of the principle investigator. All the collected data were tabulated and analyzed using Microsoft^®^ Excel^®^. The surveys that were not completed were excluded.

## Results

### Respondents

A total of 596 replies were collected; 505 women, 69 men and 23 did not answer the question (Table 1). Most of the responders were aged 25–30 years (33.2%), followed by 18–25 (26.2%) and 30–35 (21.0%) years. Fewer responses were received from responders in the > 40 years age group due to this age group being less represented among aesthetic practitioners and also less likely to be willing to participate in surveys. A total of 78% of the responders had a university education. An estimated 60% of the participants were beauty consultants and 40% dermatologists, plastic surgeons, and cosmetic doctors. The results for the assessment of each of the facial features covered by the questionnaire are described below.

**Table 1:** Patient demographics and survey question results

*n* (%)	*N* = 596
*Age group, years [n (%)]*	
18–25	156 (26.2)
25–30	198 (33.2)
30–35	125 (21.0)
35–40	32 (5.4)
40–45	11 (1.8)
45–50	26 (4.4)
50–55	10 (1.7)
55–65	2 (0.3)
N/A	37 (6.2)
*Gender*	
Female	505 (84.7)
Male	69 (11.6)
N/A	23 (3.9)
*Education [n (%)]*	
High school	58 (9.7)
University	466 (78.2)
None	3 (0.5)
N/A	70 (11.7)
*Would you have cosmetic surgery? [n (%)]*	
Yes	398 (66.8)
No	126 (21.1)
N/A	73 (12.2)
*Would your preference be for a surgical or non-surgical procedure [n (%)]*	
Non-surgical	495 (83.1)
Surgery	43 (7.2)
N/A	59 (9.9)
*Does beauty affect quality of life? [n (%)]*	
Yes	540 (90.6)
No	14 (2.3)
N/A	43 (7.2)

*N/A* no answer, *QoL* quality of life

### Facial Shape

**Fig. 1:**
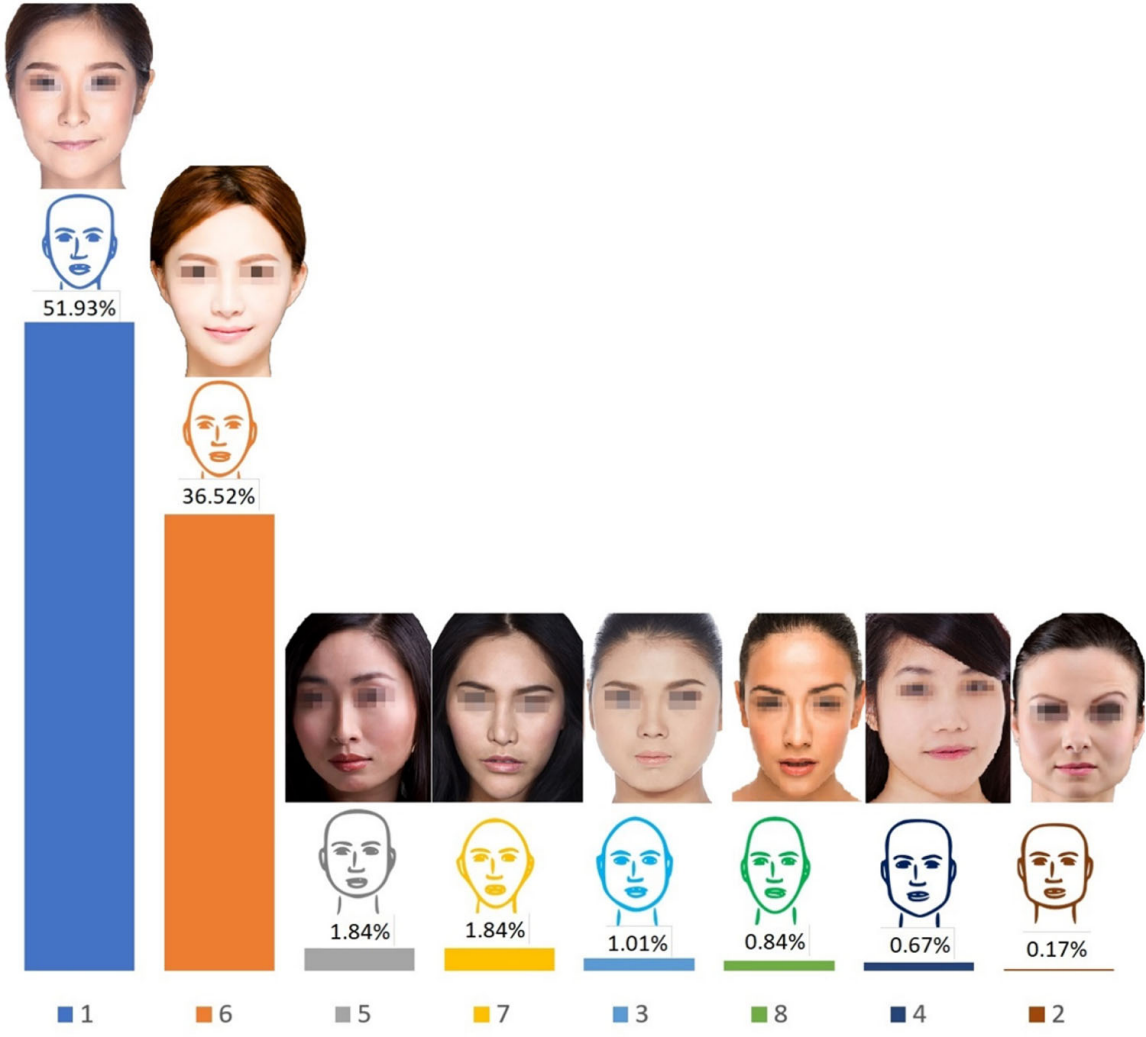
Summary of the respondents’ preferences for facial shapes. Sketches are as presented in the questionnaire. Photographs correlating to each sketch have been added for reference. Photograph credits: (1), (3), (7) Jade ThaiCatwalk/Shutterstock.com, (2) Tudor Raiciu/Shutterstock.com, (4) Kaesler Media/Shutterstock.com, (5) Geoffrey Jones/Shutterstock.com, (6) Tom Wang/Shutterstock.com, (8) Hank Shiffman/Shutterstock.com

Simple sketches representing eight facial shapes were given: heart, square, pear, rectangle, round, oval, diamond, and oblong (provided without a written description). All sketches had the same eyes, nose, and lips and no hair (Fig. 1). This was done to understand the preferences of the participants for facial shape without including gender-specific features such as hair. Four different facial shapes with pointy chins were present in this group of sketches (heart shape, rectangle, oval, and oblong). The shape most preferred by responders was shape 1 which had a narrow lower face and narrow pointy chin (51.9%), followed by the oval facial shape which was a long, thin face with a narrow round chin (36.5%). The facial shape with a wide, square lower face was least preferred by responders. Overall, the responders preferred facial shapes with a narrow lower face, and pointy round chin. Only 5% of responders did not answer this question. Figure 1 represents the preferences for facial shapes according to the responses collected.

### Female Facial Profile

**Fig. 2:**
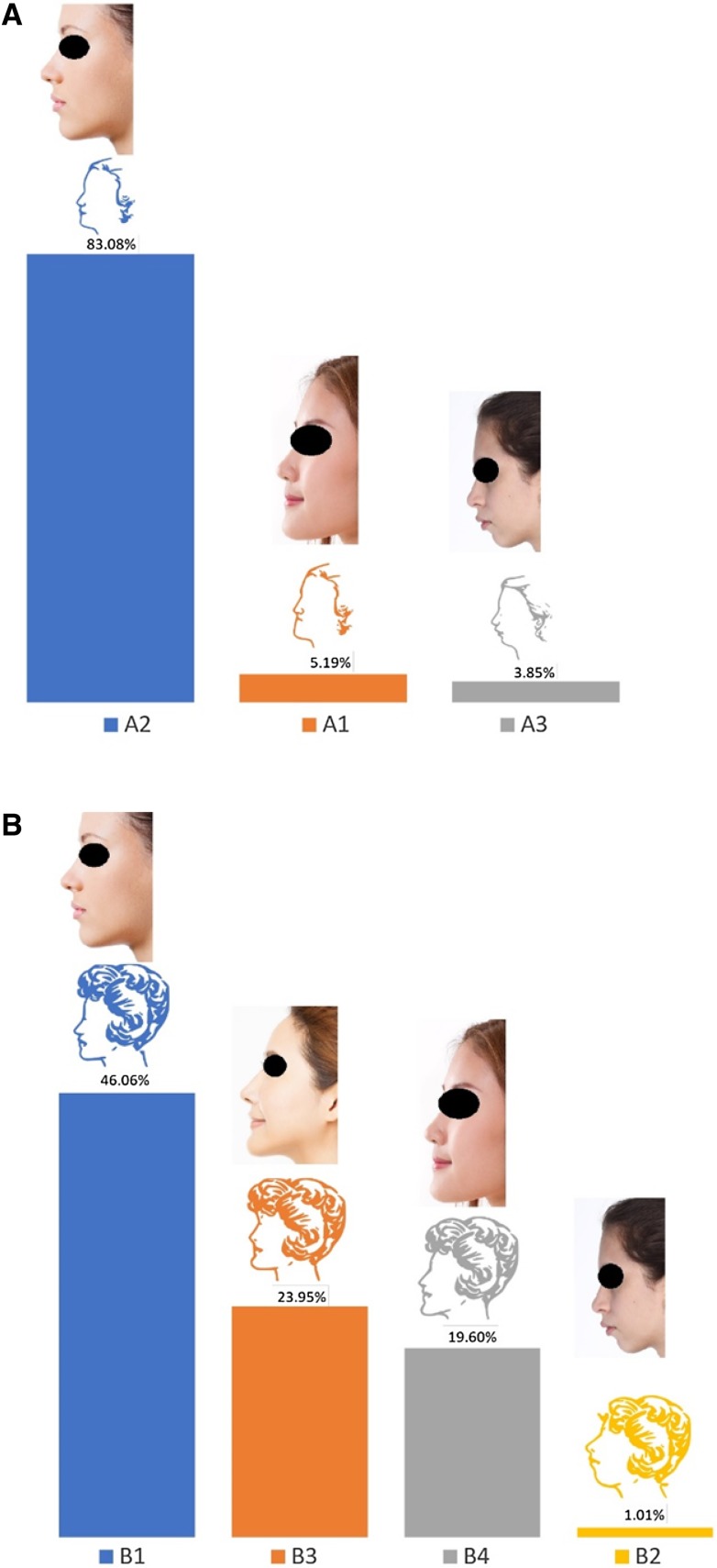
Summary of respondents’ preferences for female facial profiles. Sketches are as presented in the questionnaire. Photographs correlating to each sketch have been added for reference. Photograph credits: (B1) Valua Vitaly/Shutterstock.com, (B2) Shutterstock.com, (B3) Tom Wang/Shutterstock.com, (B4) 9nong/Shutterstock.com

Two sets of female facial profiles were given, labelled as subgroups A and B (Fig. 2a, b). The aim of the first group of photographs (A) was to present the basic skeletal pattern (Class I, Class II, Class III) and corresponding facial profiles (straight, concave, and convex). In group B, in addition to the three basic profiles, a profile with an anteriorly projected chin was added. The same hairstyle was used for all sketches in each group to remove a potential source of bias. A clear majority of responders (83%) preferred profile A2 (straight facial profile), and 46% preferred profile B1 (straight facial profile) (Fig. 2a, b). Less popular were profiles B3 (24.0%; a straight facial profile with an anteriorly projected chin) and B4 (19.6%). In general, the facial profile with a small retrognathic chin, and convex facial profile were least preferred in both groups. The majority of the participants preferred the straight facial profile, and the convex facial profile was found to be least attractive. In total, 7.9% (A) and 9.4% (B) of the respondents did not answer these questions.

### Lip Shape

**Fig. 3:**
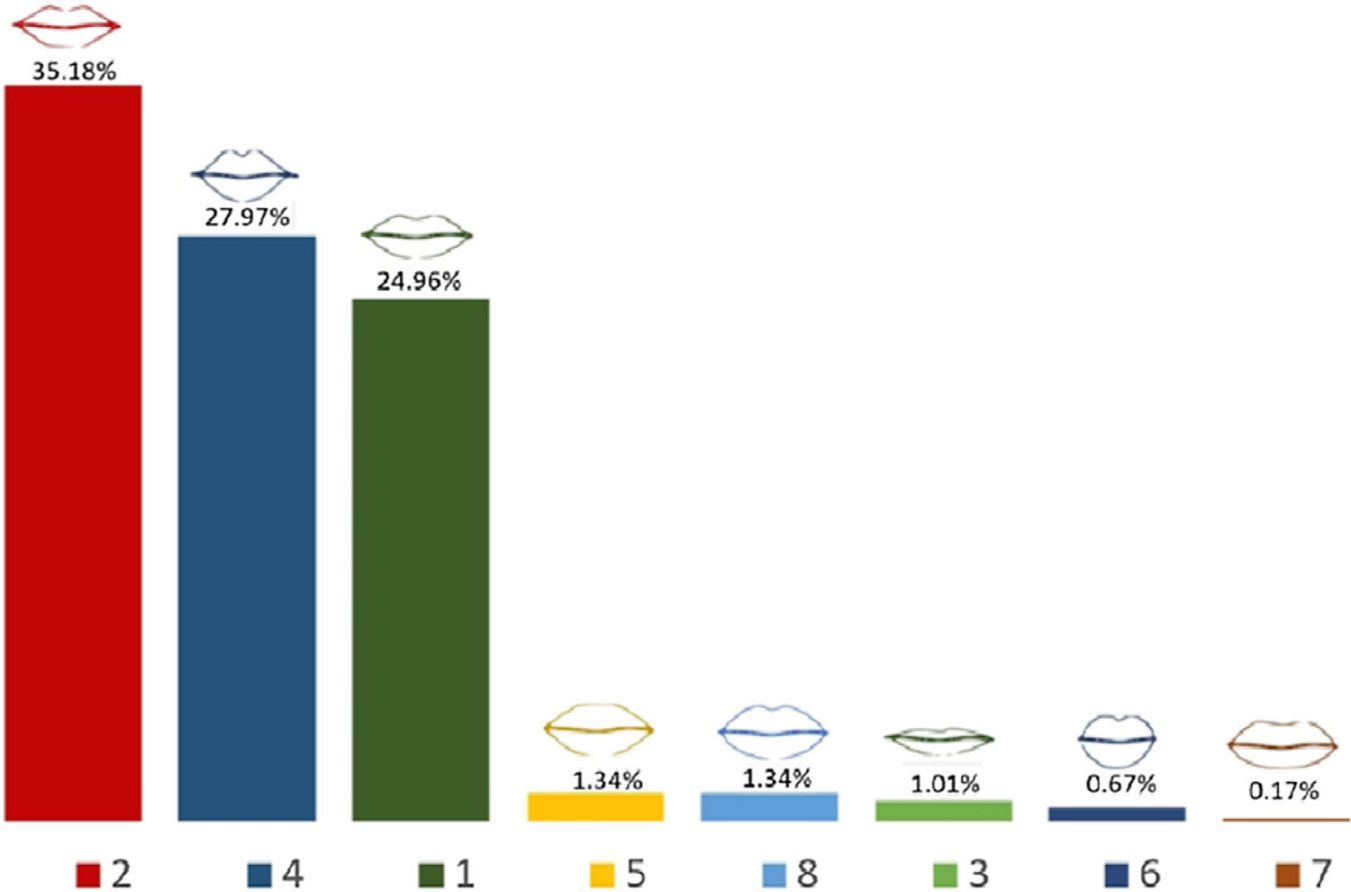
Summary of respondents’ preferences for lip shape

Eight different numbered lip shapes were presented to responders. The variations in the lip shapes included width of the lips, size of the upper and lower lips, definition of the Cupid’s bow, and variations in height-to-width ratio. Lip shapes 2, 4, and 1 were the most preferred lip shapes, 35.2%, 28.0%, and 25.0%, respectively (Fig. 3). The least preferred lip shape was lip number 7. All preferred lip shapes had a well-defined Cupid’s bow, were fuller medially and possessed less volume laterally tapering off towards the oral commissure and unequal lip volume of the upper and lower lips. In total, 7.4% of the respondents did not answer this question.

### Jaw Angle

**Fig. 4:**
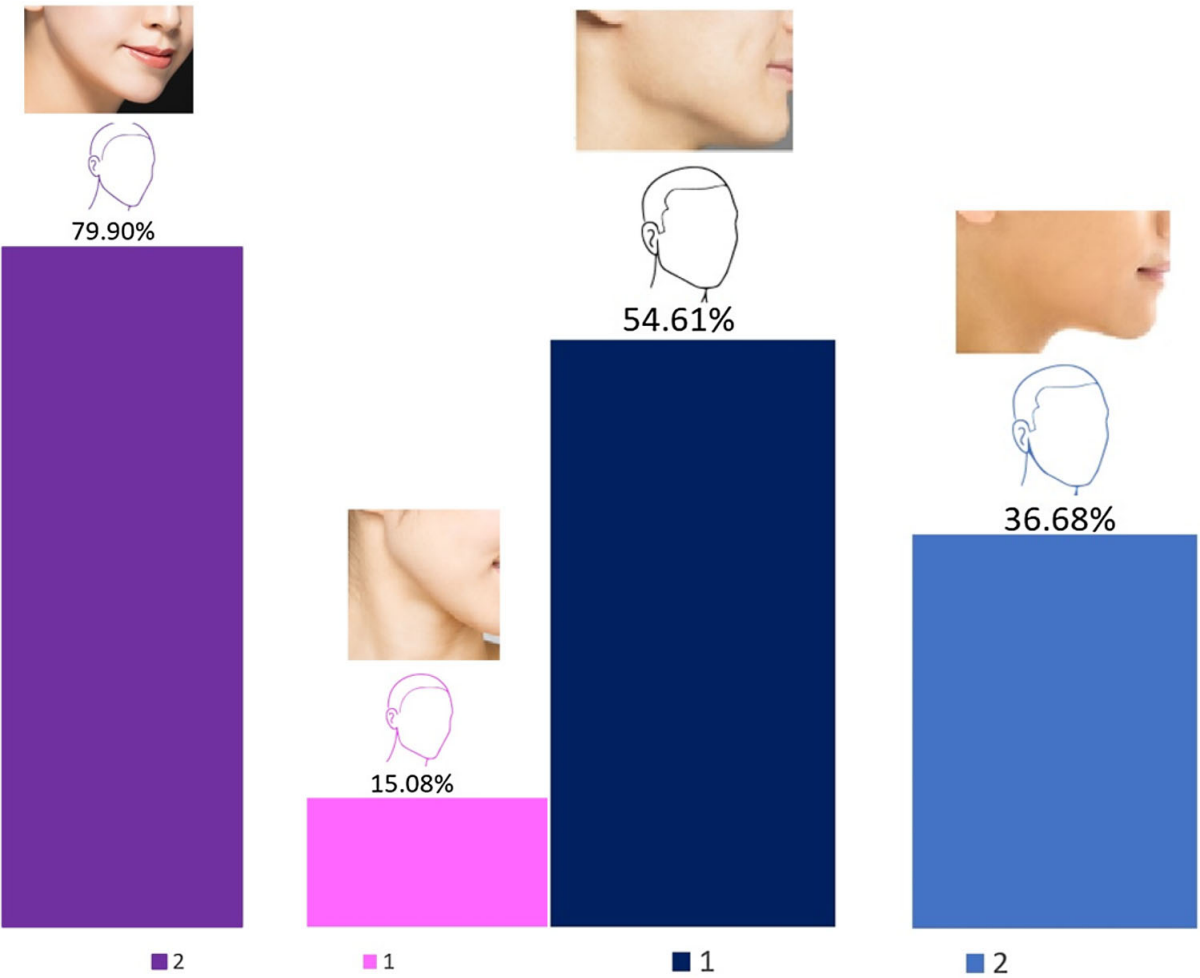
Jaw angle preferences for women and men. Sketches are as presented in the questionnaire. Photographs correlating to each sketch have been added for reference. Photograph credits: Women (1) Number One/Shutterstock.com, (2) Tom Wang/Shutterstock.com. Men (1) (2) Tom Wang/Shutterstock.com

Simple sketches for male and female faces with well-defined or obtuse jaw angles were given to respondents. The sketches were labelled as female and male. The respondents were asked to choose the jaw angle that they most preferred. A total of 79.9% and 36.7% of the respondents preferred the obtuse jaw angle in comparison with an angular well-defined jaw angle for women and men, respectively. For men, the majority of respondents (54.6%) preferred an angular and well-defined jawline (Fig. 4). In total, 5.0% (for female profiles) and 8.7% (for male profiles) did not answer these questions.

### Chin Shape

**Fig. 5:**
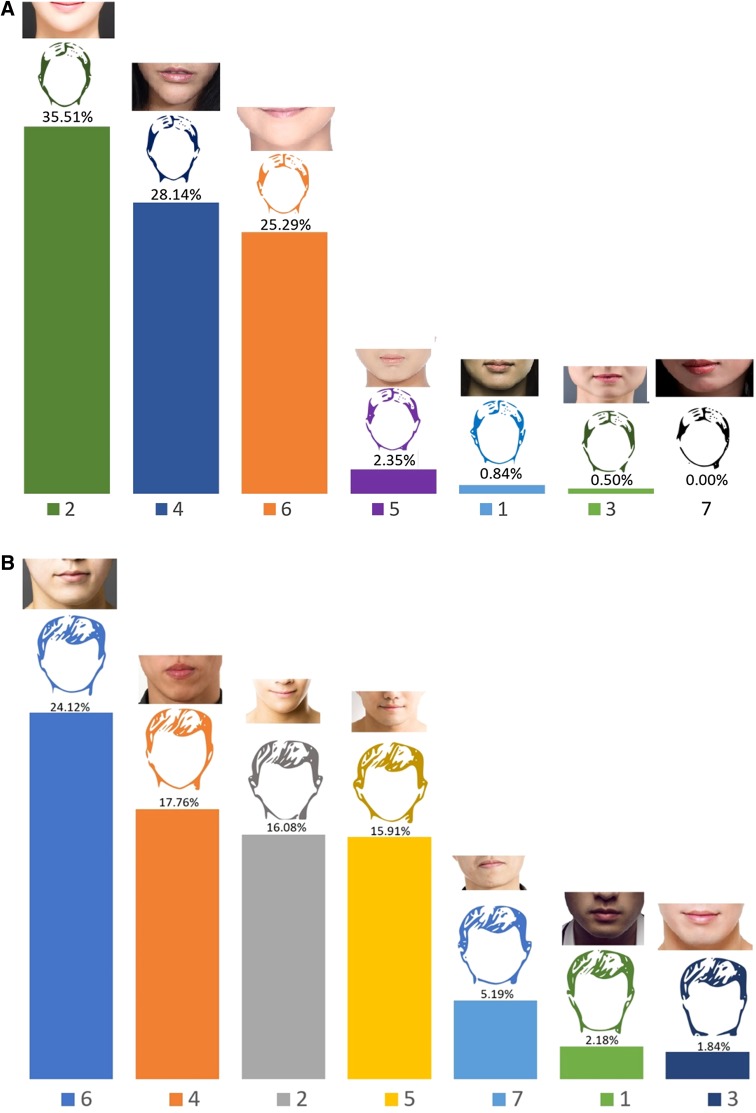
Chin shape preferences for men and women. Sketches are as presented in the questionnaire. **a** Photographs correlating to each sketch have been added for reference. Photograph credits: (1) number one/Shutterstock.com, (2) Tom Wang/Shutterstock.com, (3) Tudor Raiciu/Shutterstock.com, (4) (5) (6) Jade ThaiCatwalk/Shutterstock.com, (7) Geoffrey Jones/Shutterstock.com. **b** Photograph credits: (1) Ander5/Shutterstock.com, (2) (3) (5) (6) Tom Wang/Shutterstock.com, (4) Vladimir Wrangel/Shutterstock.com, (7) Leungchopan/Shutterstock.com

Seven different chin shapes for both men and women were presented as sketches. The same hair, upper face, and midface were used for all seven sketches. The height and width of the chin were varied between sketches. To describe them visually, options for pointy, very pointy, pointy and narrow, pointy and wide, round and narrow, round and wide, and flat chins were given (Fig. 5). Picture representations of these chin shapes have been added for easier translation of the sketches to real-life soft tissue appearance. In total, 7.4% (for female profiles) and 16.9% (for male profiles) of the participants did not answer these questions. For female sketches, the most preferred chin shapes among respondents were sketches 2 (35.5%), 4 (28.1%), and 6 (25.3%) with a narrow lower face and narrow pointed chin with a rounded or pointy apex. The least preferred chin shapes were sketches 3 and 7, with a flat and square chin and a round but somewhat flat and short chin respectively. Among the male sketches, the preferences indicated by respondents showed greater variation than for the female sketches. Sketches 6 (24.1%), 4 (17.8%), 2 (16.1%), and 5 (15.9%) all received positive assessments from respondents. All of the preferred male sketches displayed a round and relatively narrow and pointy chin. The least preferred male chin shape was the sketch with short, square lower face that displayed the squarest chin among the sketches (number 3).

### Nose Shape

**Fig. 6:**
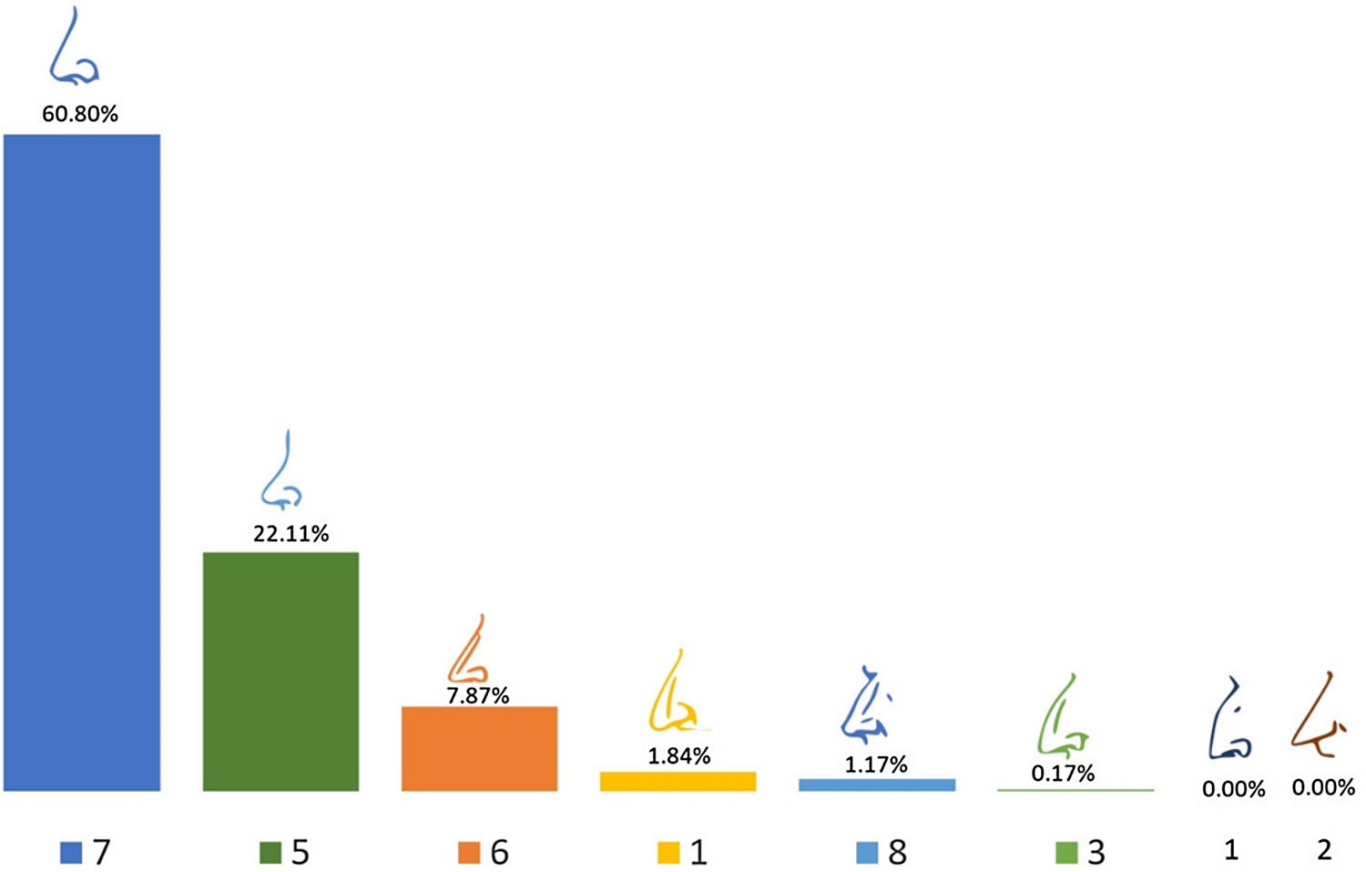
Nose shape preference

Eight sketches of nose profiles were presented which varied in the shape and projection of the bridge and tip of the nose including straight, concave, convex, and aquiline. The tip of the nose varied from straight to very pointy, varied nasolabial angle. A straight nose profile (sketch 7) was preferred by 60.8% of the respondents. This was followed by 22.2% for sketch 5 which had a slightly concave nose profile. Both of these profiles had an approximately 95° nasolabial angle. The aquiline noses and those with the tip pointing downwards were poorly rated. A nose with a preferable nose tip angle but a dorsal hump was less preferred to one with the tip pointing downwards (reduced nasolabial angle) but a straight profile. The sketches with a very straight profile, but a very pointy sharp tip and an aquiline nose (a prominent bridge, with a dorsal hump and the tip sharply bent downwards), were least preferred (Fig. 6). In total, 6.0% of respondents did not answer this question.

### Cosmetic Procedures

Three questions were asked: if the participants would consider plastic surgery, would they choose surgical or non-surgical procedures if given the choice, and whether they though being beautiful would improve their daily life. A majority (66.7%) of the respondents said they would have plastic surgery. However, if given the choice 82.9% indicated they would opt for non-surgical procedures. Finally, a clear majority (90.5%) believed that being beautiful would improve their daily life.

### Gender Bias

The data were analyzed for any bias based on responders’ age or gender. The focus of the analysis was the most preferred and second-most preferred choices for all presented questions. No gender bias was noted for facial shape, facial profiles A and B, nose shape, male and female jaw angles, chin shapes, choice of having cosmetic surgery, and the preference for non-surgical procedures. Although no gender bias was noted for lip shapes 1 and 2, it is worth noting that lip shape 4 was preferred by more females than males.

### Age Bias

The most preferred facial shapes were facial shape 1 and then facial shape 6. As a general trend, with age the preference for facial shape 1 decreased and for facial shape 6 increased. No age bias was noted for facial profiles A; however, facial profiles B showed age bias. With age the preference for facial profiles B3 and B4 that have more anteriorly projected chin increased. The younger age groups predominantly preferred facial profile B1 (straight) followed by B3 (anteriorly projected chin) with an increased preference for B3 with age. Although the preference for nose shapers 5 and 7 was similar for all age groups, 100% of the age group 55–65 preferred nose shape 7. The preference for lip shapes 1, 2, and 4 was the same across all age groups apart from 40 to 45 age group who had a strong preference for lip shape 1 and age group 55–65 who preferred lip shapes 2 and 3 with a 50% ratio. In comparison with other age groups, the age group 55–65 had 100% preference for an obtuse jaw angle for women. No gender bias was noticed for the male jaw angle, male and female chin shape, and the option for surgical or non-surgical procedures. The age groups 30–45 had responded more positively to the question regarding having cosmetic surgery.

### Comparison of Ideals of Beauty Among Aesthetic Professionals and Laypersons

A comparison of the most preferred facial profiles in this study and those of the previous study conducted in Chinese laypersons is presented in Table 2. Overall, this survey revealed that Chinese aesthetic practitioners preferred a heart/inverted triangle facial shape with a reduced lower face height, a straight and small nose, as well as lips that are full medially and taper off laterally with well-defined borders and Cupid’s bow. The aesthetic practitioners’ second-most preferred facial shape was the layperson’s first preferred facial shape [8]. Furthermore, the aesthetic practitioners preferred a narrow pointy chin for women in contrast to the laypersons who preferred a narrow, but less pointy chin. Differences in preference of jaw angle were also observed, with an angular jaw angle preferred by the aesthetic practitioners, in contrast to laypersons whose preference was divided between the angular and obtuse jaw angle, neither preference dominated the other strongly. Finally, the preferred lip shape among aesthetic practitioners also varied from the preference of the laypersons; a narrower lip with medial fullness tapering laterally was the first preference among the laypersons, whereas aesthetic practitioners preferred wide, less volumized lips, with less acute tapering laterally [8].

**Table 2:**
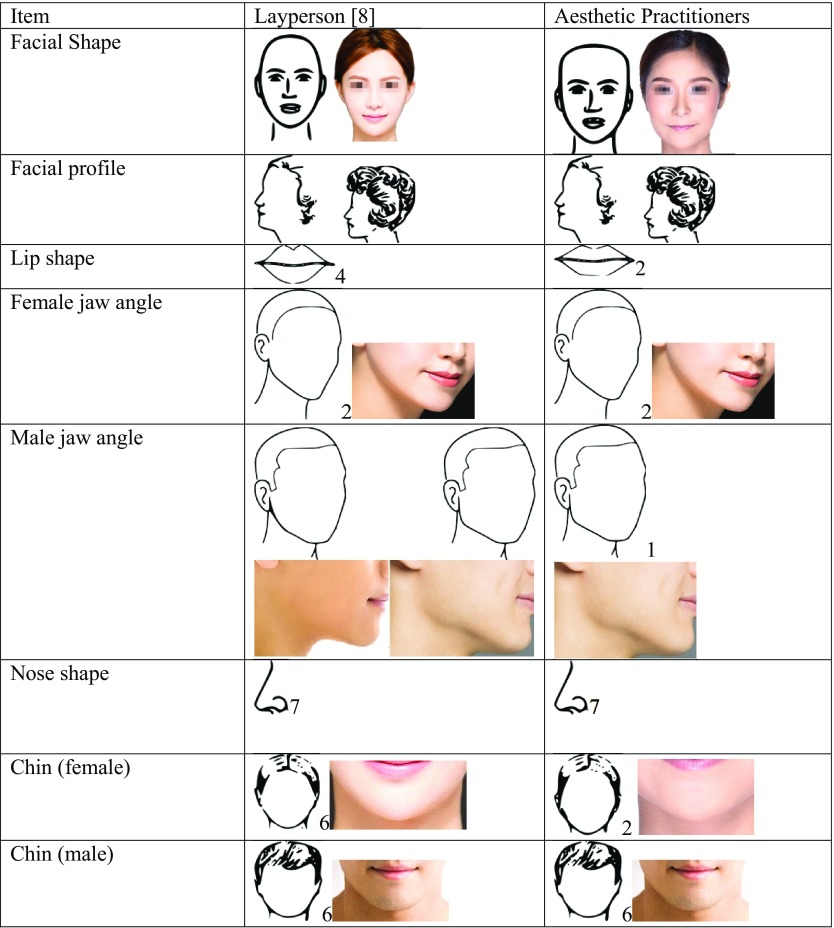
Differences between preferences for various facial features between laypersons and aesthetic practitioners. Sketches are as presented in the questionnaire. Photographs correlating to each sketch have been added for reference

Photograph credits: Facial Shape; (6) Tom Wang/Shutterstock.com, (1) Jade ThaiCatwalk/Shutterstock.com, Female jaw angle; (2) Tom Wang/Shutterstock.com, Male jaw angle; (1) (2) Tom Wang/Shutterstock.com, Chin (female); (6) Jade ThaiCatwalk/Shutterstock.com, (2) Tom Wang/Shutterstock.com, Chin (male); (6) Tom Wang/Shutterstock.com

## Discussion

Treatment strategies for aesthetic medicine need to be considerate of facial morphology, cultural and geographic ideals of beauty, and what individual patients deem attractive. For optimal treatment planning and treatment outcomes, clinicians must understand, acknowledge, and address not only the commonalities, but also the variations in ideals of beauty among their patients [9]. In addition, between each individual patient and between various ethnicities there are unique facial features and bone structures that significantly influence the outcome of any cosmetic procedure. Using generic treatment templates without taking individual characteristics into consideration, or not understanding the ideals of beauty of individual patients, could result in suboptimal outcomes and low levels of satisfaction. The aim of this study was to examine aesthetic practitioners’ ideals of beauty and their preferences for facial shapes, facial profile, nose, lips, jaw angle, and chin shape. This was then compared to a previous similar previous study in which the same ideals of beauty were investigated among Chinese laypersons. These results enable analysis of the differences and similarities of ideals of beauty between these groups, which may help further aesthetic practitioners’ understanding of their patients’ needs and expectations. To the author’s knowledge, there have been no previous studies or research into practitioners’ own perception of beauty and the potential influence this may have on treatment options offered to patients.

Clinicians offering cosmetic procedures should understand the aesthetic ideals of their patients, as well as cultural and patient-specific preferences. Being aware of cultural differences goes beyond understanding a patient’s language or facial characteristic differences, and it also involves an understanding of what patients desire and how they want to enhance their natural beauty. Key components of a successful consultation and treatment planning include understanding patients’ ideals of beauty and expectations as well as the practitioners’ own ideals of beauty perceptions about the patients’ expectations and ideals of beauty. In this regard, the results of this study revealed that ideals of beauty are largely the same between Chinese aesthetic practitioners and laypersons. However, differences were observed in the preferences for facial shape, lip shape, male jaw angle, and female chin shape. An oval facial shape with a smooth flow from the forehead through temples, zygoma and cheeks, jaw angle and jawline, and the chin has been reported to be attractive in all cultures [4, 10, 11]. Ahn et al. [12] reported that a delicate ovoid facial shape is favoured in Asia. Similarly, Park et al. reported that “oval and almond-shape faces” are preferred by Asian women [13]. A study by Chen et al. [14] also reported on a preference among Asian people for female faces which are “elliptical” as it is considered to be feminine and gentle. It was therefore interesting to find that a heart-shaped face with a narrow and pointy chin was most preferred by the aesthetic practitioners. This could be due to an exaggerated move towards the influence of “Korean Pop” faces, and the “V-shaped” face. However, this preference was not shared by the laypersons, as their most preferred facial shape was the oval facial shape [8].

This study also found that aesthetic professionals preferred an obtuse jaw angle for women and a square well-defined jaw angle for men, and a round and pointy chin for both women and men. In contrast, the previous study of ideals of beauty among Chinese laypersons revealed a preference for an oval facial shape with a round but not pointy chin and many respondents preferred an obtuse jaw angle [8]. This finding is in agreement with previous reports that Asian patients dislike a square jaw as it makes the face look wider and disturbs an oval appearance [13]. A square jawline is considered masculine, and Asian women find it “aesthetically displeasing” [11, 14, 15]. In addition, cultural beliefs mean that in Asia, a person with square jawline can be thought as “stubborn or even ill fated” [16]. Interestingly, Park et al. [13] also reported that most patients requesting facial contouring procedures to reduce the lower face width are between 20 and 40 years of age.

The Asian nose is usually small, with a flat bridge, and wide nasal tip [17–19]. The enhancement and improvement of these features are commonly requested procedures among Asians, in particular in China and South Korea [17, 19]. The desired outcomes are improved nasal bridge height, improved tip projection, improved profile, and nasolabial angle [17, 19]. These are in line with our findings among the laypersons and aesthetic practitioners. Nose shape 7 with a flat straight nasal bridge and a well projected tip was the most preferred sketch in this study and among laypersons [8].

These results are important given that a clinician’s own perception of beauty affects their assessment, judgement, and treatment planning. For example, a consultant or a clinician who deems a strong jawline (square facial shape) as non-feminine, will offer patients options to reduce their lower face width. In comparison, a clinician who is not familiar with Asian ideals of beauty and follows the trends of an “Angelina Jolie jaw angle and jaw line” for women, would find the obtuse jaw angle in Asian women unattractive and suggest enhancement. At a recent cosmetic congress in Asia, the author observed a non-Asian doctor enhancing the jawline of a female Asian volunteer model from obtuse to a square jaw angle with 3–4 mls of dermal fillers on each side during the main agenda. The doctor explained and believed women desire well-defined “Hollywood style” square jawlines. However, the volunteer model, in line with ideals of beauty in Asia, had her jaw angles and masseters treated previously to reduce the width of her lower face and create an obtuse jaw angle. Indeed, in Asia and China, facial features are considered to be a representation of one’s fortune and certain features are believed to bring about luck or good fortune and vice versa [20]. The mandibular angle is an example of this belief and plays a very important role in female facial shape in Asia: “a woman who has a wide and square face is thought to have had a unhappy life” [21]. Accordingly, surgical and non-surgical reduction in the lower face width is a common request and a very popular practice in Asia [12, 15, 22, 23].

The findings of this study support several previous studies that also reported a straight facial profile is the most preferred [24–26]. In Soh et al.’s study, a straight profile (normal or bimaxillary retrusion) was perceived to be most attractive for both genders by their participants (orthodontists, dental students, and laypersons in the Asian community) and mandibular prognathism the least attractive by all three groups [24]. This was consistent with the present study’s findings. However, an interesting finding of this previous study was that the dental professionals ranked bimaxillary retrusion in female profiles as more attractive than laypersons and dental students. This result shows that dental professionals consider the bimaxillary retrusion facial profile for female patients an ideal post treatment profile whereas the laypersons may consider such a profile only acceptable. This further highlights the differences between the ideals of beauty among laypersons and healthcare professionals which could result in overcorrection of Chinese facial profiles.

This survey revealed several important characteristics of Chinese aesthetic professionals. Firstly, the majority of the aesthetic practitioners surveyed indicated they would be willing to have plastic surgery and 82.9% of this total would opt for non-surgical procedures if given the choice. Interestingly, the results of this survey also revealed that, in comparison with laypersons, aesthetic practitioners were more likely to undergo plastic surgery. This result is valuable given that studies on the attitudes of aesthetic practitioners towards surgery are not readily available. A clear majority of aesthetic practitioners surveyed (90.5%) also believed that being beautiful would improve their daily life which was a larger percentage in comparison with the laypersons surveyed in a previous study.

In terms of demographics, 84.6% of the aesthetic practitioners who responded to the questionnaire in this study were female suggesting that in China aesthetic medicine is a relatively female-dominated industry. This study found that younger consultants and doctors were more likely to answer the survey. The > 40 years age group were present in smaller numbers and were less likely to complete the survey. This could be due to cultural aspect of these practitioners. In China, the concept of keeping “face” and an “older”, “professional” “more experienced” status in front of junior colleagues is still alive and important and may present a barrier to participating in surveys. Also, the importance of keeping “respect for age and rank”, and maintaining composure may explain the relatively smaller number of responders in the > 40 years age group [27]. Moreover, prior to the economic reforms, cosmetic surgeries were not popular or performed due to cultural believes. The first generation of plastic surgeons (focusing on reconstructive surgery and not cosmetic surgery) were trained in 1920s and 1940s and the emergence of cosmetic clinics in 1930s. During the cultural revolution’s 10-year period, plastic surgery suffered massively in China and was reported to suffer “complete demolition” with a few reconstructive surgery departments remaining functional. During Maoist era, the communist ideology emphasized on “iron women” with plain blue dresses/unisex blue trousers and worker jackets, short hair, and no makeup. Plastic surgery was labelled “bourgeois vanity”. As late as 1960s and 1970s, plastic surgery for purely cosmetic reasons was prohibited apart for some special cases (e.g. actresses) and continued until 1980s. Thus, reduced number of plastic surgeons with the speciality only being revived only in 1980s and mainly peaked in 2000s [28, 29]. In conjunction, until 2000, less than 1% of China’s households had the income to support elective cosmetic procedures [30]. It is therefore not a surprise to see a relatively young distribution of aesthetic practitioners including aesthetic doctors, dermatologists, and plastic surgeons with the market starting to emerge in the early 2000s.

Beauty consultants who are mainly female and young, play a key role in facial assessment, treatment planning and product/device selection and recommendation to the patients. They carry out facial assessments on patients, offer treatment plans, and suggest treatment products or devices. The patient consents to the provided treatment plan by the beauty consultant, pays for the procedure, and then meets with the doctors who are performing the procedure.

In China, doctors who focus on injectables are known as “injector doctors” and can see up to 40–50 patients a day [31]. For many aesthetic practitioners (injector/dermatologist/plastic surgeon), they may have less than 10 min to perform the planned procedure. Furthermore, on average, a busy plastic surgeon can see up to 10 patients per day for oculoplastics, or more than 5 for rhinoplasty with numbers increasing during summer vacation and before the Chinese New Year [31]. As such, there is limited opportunity to carry out a comprehensive consultation, assessment, and treatment planning. The beauty consultants’ ideals of beauty are therefore a strong influencer on the patient decision, more so than the treating physician, and the most likely outcome for the attending patients. “Successful” consultants are very highly regarded by Chinese cosmetology hospital managers and owners, majority of whom are business men/women and not healthcare professionals. This role has been developed to help both large and small cosmetic clinics and hospitals in China due to the large number of patients. Therefore, it is important to understand the ideals of beauty among Chinese beauty consultants in addition to aesthetic doctors and surgeons as they have a large influence on patients and treatment plans.

Although interpretation of some of the results must to be done cautiously, the age of the aesthetic practitioners, their level of experience and expertise, and knowledge of the aesthetic trends may affect their ideals of beauty.

There is a difference between patient-centeredness and doctor-centeredness. Patient-centeredness describes the attitude that is shared by both doctors and patients where both parties have a willingness to share information and plan care collaboratively. Doctor-centeredness represents a situation where doctors take the lead in handling information and planning care [32]. Patient-centeredness has been shown to result in enhanced patient satisfaction, and it is beneficial for both patients and doctors to have similar attitudes [33]. Multiple studies have shown that the perception of aesthetics and aesthetic treatment needs show differences between patients and the healthcare professionals (dentists) [34–36]. As such, differences between perceptions of beauty and ideals of facial beauty may influence the decision-making process. In this study, some of the ideals of beauty for certain facial features showed variation between laypersons and aesthetic practitioners. In countries like China, where the consultation and treatment planning are carried out by “consultants/beauty consultants”, and not the treating physician, this is of particular importance. In addition, this misalignment in ideals of beauty among healthcare practitioners and the patients has the potential to result in patient dissatisfaction.

This study had several limitations which should be mentioned. Firstly, actual photographs were not used in the questionnaire and only sketches were provided, which may mean the results do not exactly match with the respondents’ perceptions of real human faces. However, this decision was made to eliminate bias, as if presented with a photograph of a real human face features or characteristics other than those being studied may have influenced the respondents’ assessment of the face. In addition, the study did not investigate the link between ideals of beauty and clinical practice; however, this would be an aim for future research. The small number of responders in the age group > 40 would mean the results for this age group may not be statistically significant. Finally, studies based on survey data have inherent limitations including a lack of longitudinal data and the potential for differences between respondents’ answers on a questionnaire versus how they would behave in real clinical practice.

## Conclusion

The aim of this study was to understand Chinese aesthetic practitioners’ perception and ideals of beauty which may be directly related to clinical assessments, facial analysis, and possibly treatments offered. In addition, understanding aesthetic practitioners’ ideals of beauty is also important during doctor–patient communication for strengthening practices of patient-centred communication. This study showed that there are differences in the ideals of beauty between laypersons and aesthetic practitioners. Understanding these will help the aesthetic practitioners to move towards patient-centeredness, where both doctors and patients have a willingness to share information and plan care collaboratively, resulting in enhanced patient satisfaction. The practitioners who work in the field of aesthetics and aesthetic medicine must recognize the variability of cultural identities and ideals, wishes and desires, attitudes, concerns, and uncertainties of each individual patient.
